# How medical teachers use narratives in lectures: a qualitative study

**DOI:** 10.1186/s12909-015-0498-8

**Published:** 2016-01-07

**Authors:** Graham Easton

**Affiliations:** Imperial College, London, UK; Institute of Education, London, UK

**Keywords:** Undergraduate, Medicine, Lectures, Narrative, Learning theory

## Abstract

**Background:**

There are strong theoretical arguments for using narratives in teaching and learning within medicine, but little is known about how they are used in medical lectures. This study explores the types of narratives lecturers use, the attitudes of lecturers and students to the use of narratives in teaching, and the aspects of learning that narratives may facilitate.

**Methods:**

Observation of three medical lectures was followed by one-to-one interviews with the respective lecturers, and separate focus group interviews with medical students who attended each of the three lectures.

**Results:**

Lecturers used a variety of narratives on a range of themes, from clinical cases to patient experience narratives or narratives about their professional careers. Students and lecturers highlighted key aspects of narrative learning: for example providing a relevant context, as a “hook” to engage the audience, and as a memory aid.

**Conclusion:**

The findings support existing literature which suggests that narratives may be a useful tool for learning in medicine. This study suggests that narratives tap into several key learning processes including providing a relevant context for understanding, engaging learners, and promoting memory. For medical students in lectures, narratives may be particularly relevant in promoting humanistic aspects of medicine, including professional identity and empathy.

## Background

There are strong pedagogical and theoretical arguments for the benefits of narrative learning (learning through narratives, or stories) in medical education—particularly in the areas of meaning-making (“making sense”) [1], the development of identity [2], enhancing memory [2–4], promoting empathy [5, 6], reflection on practice [7], and the development of clinical reasoning through what are known as illness scripts [2, 8]. All these areas are pertinent to training doctors, which suggests that using narratives as a teaching tool may be a powerful approach in medical education. Although most undergraduate medical courses around the world still use the traditional lecture format extensively within their curricula [9], there is very little information about the extent to which lecturers use narratives, how they use them and what impact this may have on student learning.

**Table 1:** A typology of narratives used in medical lectures

Narrative theme	Description	Examples	Dominant aspects of learning process
Clinical case narrative (hypothetical)	Narrative about a hypothetical “typical” clinical case	• a typical patient with a drug overdose in the Accident and Emergency department	Meaning-making Relevant context Engagement Memory Professional identity
Clinical case narrative (real)	Narrative about a real clinical case	• side effects the lecturer’s wife experienced after using opioids	Meaning-making Relevant context Engagement Memory Professional identity Promoting empathy/compassion
		• a lecturer’s experience of a friend’s mental illness	
History of medicine [discovery]	Narrative telling story of important medical/scientific discovery	• story about the introduction of laudanum into the UK in the 1600’s	Meaning-making Relevant context Engagement Memory
History of medicine [social]	Narratives about historical society’s relationship with medicine	• recent abuse of Fentanyl patches in Canada	Meaning-making Relevant context Engagement Memory
		• how chlorpromazine revolutionised the treatment of schizophrenia	
Patient experience narrative (distant)	A narrative about a patient’s experience of illness. The patient is not a patient of the lecturer.	• Story about patients who wake up every few minutes with sleep apnoea, using the analogy of having an alarm clock going off every few minutes	Promoting empathy/compassion Professional identity Memory
Professional autobiography	A narrative about lecturer’s own professional career	• why lecturer became interested in studying breathing during sleep	Professional identity Memory
Real world narrative (hypothetical)	Narrative relating basic science to real-world experience, relevant to audience but not a “real” story	• narrative explaining pain suppression pathways by referring to the experience of a sporting injury	Meaning-making Relevant context Engagement Promoting empathy/compassion
Scientific process narrative	Narrative about a natural process, and/or how science has manipulated it to its advantage *[often use analogy or metaphor]*	• narrative approach to describe a slide showing the sequence of steps involved in the brain inhibiting pain pathways	Relevant context Engagement Memory
Social impact of medicine	Narrative primarily about the social impact of medical science or medicine [contemporary]	• the drug Quetiapine and the financial implications of pharmaceutical marketing	Meaning-making Relevant context Engagement Memory

**Fig. 1:**
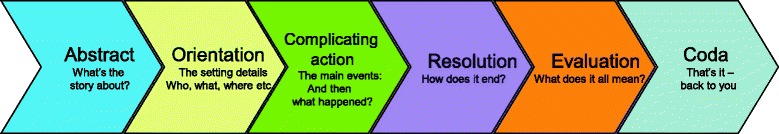
Labov’s sociolinguistic model of personal narratives (adapted) [28].

Narrative learning theory is an attempt to explain how narratives might promote learning. The central proposal for narrative learning theory is that:

“*The frames of meaning within which learning occurs are constructions which grow out of our impulse to emplot or thematise our lives*” ([10] p10).

Narrative learning is based on the premise that an effective way to convey educational messages is via these constructions: learners connect new knowledge with lived experience and weave it into existing narratives of meaning [11]. In other words, we have a natural predisposition to make sense of our experiences and actions by “storying” them, or creating coherent narratives from the chaos of life. Narrative learning theories fall under the broad umbrella of constructivist learning theory [12], which understands learning as construction of meaning from experience. There are clear parallels between narrative learning theory and the dominant theory of learning in adulthood—experiential learning theory; the idea that adult learning is integrally related to lived experience [13]. Clark and Rossiter argue that experience is always pre-linguistic—it has to be turned into narrative form, or “storied” before the learner can make meaning from it [14]. Stories have been proposed as potentially powerful educational tools because they are believable (often making the unfamiliar familiar), memorable, and engaging [15]. Narratives demand that listeners become involved in creating meaning which can promote memory, and characters and their motivations encourage an emotional response that facts alone cannot [11].

There is evidence that stories are used widely outside the lecture theatre in medical training. Formal case histories (professional narratives about clinical cases) are used widely, for example on ward rounds or in meetings, and observational studies have confirmed that more informal “anecdotes” (which Hunter describes as “informal stories of clinical cases, solved and unsolved”) are used widely in educational settings in hospitals [2, 16]. A questionnaire study has suggested that GP trainers also use anecdotes frequently in teaching trainee GPs in a clinical GP setting [17]. There are many other examples of narratives being used in the curriculum, for example in medical humanities programmes [18], significant event analysis (or critical incident analysis) [19], and reflection on practice [20].

However, research is lacking on the use of narratives in basic medical science lectures—where perhaps the more formal and traditional setting encourages a more didactic teaching style. Although lectures may be as effective as any other teaching method for delivering information [21], there is little evidence that they have any role in modifying behaviours or values or inspiring interest, and there is a danger that learners can be overwhelmed by a large volume of information for which they have no context [9]. Educational theory suggests that tactics such as setting memorable context, or encouraging the development of relevant connections or anchors may help students to get the most from their lectures [22]. Stories could be an effective way to promote understanding and embed new ideas in the narrative component of the listener’s long-term memory [23].

There have been two attempts to develop a “typology” of narratives used in medical education. One outlines a theoretical structure for the use of narratives in higher education, using examples from nurse education [24]. The other typology is based on the types of narratives used by surgeons in training surgical trainees in the operating theatre [25]. A literature search found no attempt to describe the types of narrative used in the lecture setting.

In summary, there are ample theoretical grounds for the use of narratives in teaching and learning medicine, and evidence that they are being used in hospitals and in GP surgeries. But there is little information on how lecturers use narratives in their basic science lectures to medical students; what types of narrative lecturers are using, how narratives might promote learning, and what students and lecturers think of narratives as a teaching tool.

This study aims to identify and categorise the narrative themes that medical lecturers use in teaching (a typology), and to identify the different types of learning, or parts of the learning process, that may be facilitated by using narratives. In addition it explores students' and lecturers’ perspectives on the use of narratives and stories in medical lectures.

## Methods

The author has adopted a flexible, qualitative research design, compatible with a constructivist epistemological position. The study used a multi-method approach to data collection which includes:

- Non-participant unstructured **observations** of lectures (three in total)
- **One-to-one interviews** with the lecturers (three in total)
- **Focus group interviews** (three) with a total of thirteen students who attended the lectures (three)

### Observation of lectures

The author observed three basic science lectures, which each lasted 45 min to an hour. Direct observation allowed for exploration of how lecturers were using narratives in their lectures; in particular what narrative themes, or types, they used (the typology) and audience reactions.

### One-to-one semi-structured interviews with lecturers

One-to-one semi-structured interviews with the three respective lecturers were guided by a semi-structured interview guide . They explored their perspectives on the use of narratives in general, their views on the author’s analysis of their lecture in terms of narratives they told, their views on a few specific narratives and the meanings they were intending to convey, and their potential role in teaching and learning. The three interviews lasted between 40 min and 1 h. The interviews were based on a mixture of key questions with subsequent items depending on responses, some suggestions for prompts including reading them relevant passages of their lecture in which they used narrative, and used mainly open-ended questions (see question quide).

### Focus group interviews with students

The author facilitated three 1-hour focus groups each discussing one of the three lectures. This allowed for an exploration of both individual and collective perspectives on the meanings attributed to narratives within lectures, and in more depth than questionnaires or surveys alone [26]. The interviews were guided by a semi-structured interview guide, designed to explore students’ thoughts on how any stories might have influenced their learning.

Audio recordings—of all lectures, interviews and focus groups, were transcribed by a professional transcribing service and uploaded to NVivo software for analysis.

### Sampling

The study used a mix of purposive and convenience-based sampling. It was important to observe basic biomedical science lectures which were likely to be rich in narratives or stories, as those were the focus of the observations. In addition—using the convenience approach—the author needed to observe lectures between February and June 2013 in order to collect data in time for analysis and write up by the project deadline. On this basis, the author approached three lecturers to observe their lectures:

- **Lecture 1**: Respiratory system (to approx 400 first-year medical students on undergraduate MB BS programme)
- **Lecture 2**: Pharmacology and therapeutics (to 50 first-year medical students on graduate entry programme)
- **Lecture 3**: Pharmacology and therapeutics (to (the same) 50 first-year medical students on graduate entry programme)

Sampling for the student focus groups was purposive to explore the perspectives of students who had attended the lectures, so the groups were invited from those cohorts via sign-up sheets after the lectures. The sign-up sheet and consent forms explained that the study would explore the teaching approaches used during the lecture with neutral reference to the use of narratives. Approximately 20–30 students signed up from each lecture. For lecture 1, the final group consisted of four males and three females; for reasons related to student availability, focus groups for lectures 2 and 3 included the same participants as they had attended both lectures (four males and two females). Students were offered refreshments and a £10 Amazon voucher as reasonable compensation for their time (agreed by ethics committee). The author did not know and had not taught any of the students in the focus groups. One of the three lecturers was a colleague of the author.

### Data analysis

Narrative enquiry was the basis for analysis. For the first phase of analysis, the author *identified* narratives from within the data, and derived *categories* inductively from the raw data [27]. To identify narrative “units” the author used Labov’s model of the structure of the personal experience narrative, developed from his sociolinguistic work on the varieties of English [28]. (See Fig 1).

After identifying narratives in this way, a thematic analysis approach was used to code them according primarily to narrative content or theme and then, after lecturer and student interviews, to possible constructed meanings (of which there were often several for each narrative). Using an analytic inductive process [29], the author then applied this knowledge to observations of further narrative examples both within the same lecture, and in different lectures.

The author’s interpretation of the “meanings” within each narrative was informed and refined through analysis of interviews with the lecturers, and focus groups with students.. In this way, the author’s interpretation could be checked against theirs, and in later analysis of the interview transcript, their comments and interpretations could be linked to the narrative in question.

In addition, the author asked lecturers and students about their perspectives on the use of narratives in general, and analysed those in a similar inductive process, developing key themes around perspectives of use of narratives in science lectures.

### Ethical approval

The project received ethical approval from both Imperial College Medical Education Ethics Committee and the Institute of Education ethics committee, and written informed consent was obtained from all participants to participate in the study and for a report to be published using anonymised data.

### Scientific rigour

To minimise threats to validity in description, audio recordings of lectures and interviews were used, and field notes of key moments and time logs were kept. The author has tried to adopt Mason’s approach to maintaining validity in the area of interpretation—demonstrating how the interpretation was reached and justifying the steps through which he made his interpretations [30].

Reissman suggests that traditional ideas about reliability and validity do not apply to narrative analyses but she outlines four measures through which we can make claims for thetrustworthiness of narrative interpretations [31]. These are: persuasiveness (when the interpretation seems convincing, often because the theoretical claims are supported by participants’ accounts), coherence (where the overall goals of a narrator seem to match more detailed linguistic devices, or where key themes seem to repeat themselves throughout narratives), pragmatic use (where a study is cited or used by others), and correspondence (where researcher takes back results to those who have been studied). Of these, the author hopes to show that the findings are persuasive by clearly matching theory to data, and that themes he has recognised are persistent within narratives and across participants. There was also extensive use of correspondence by discussing lecturers’ views with students and vice versa, and by asking both types of participant their views on the author’s analysis of the narratives told during the lectures. During interviews and focus groups, the author presented some examples of narratives he had identified to lecturers and students and asked them whether they agreed this was a type of “story”. The author then went on to ask what lecturers’ intended meanings were, and what students’ understanding of the stories was. Further questioning explored the lecturers’ reasons for telling the story, why it was told in a certain way, and whether students found it helpful for learning and if so in what ways. Finally, the author would reveal his own analysis of the story in question, describing its place within the constructed typology—its dominant theme—and ask students and lecturers if they felt that this analysis made sense to them.

## Results

Nineteen narratives were identified (of which four were classified as “stories”; see glossary) in the first lecture; eleven narratives (of which three were “stories”) in the second lecture; and eighteen narratives (of which three were “stories”) in the third lecture.

Whilst there were several character-driven stories told during the lectures, they only accounted for ten of the forty-eight narratives identified across all three lectures; roughly 20 %. One example was the story of a person travelling on the tube, used to introduce the different phases of sleep:

### Lecture 1

*The first bit is Stage I and Stage I sleep is that drowsy stage which you can observe in a practical laboratory called the tube. So if you watch somebody on the tube, you’ll see them start to lose that postural muscle activity as they fall asleep. And their head goes (lecturer pretends to nod off to sleep) and you’ll also see them lose their ocular eye muscle activity and their eyes will start to roll—they’ll do this (lecturer rolls eyes). But in Stage I sleep you’re still responsive to sound, so when he gets to South Kensington you’ll see them do this as they sit up and then as you go through the different stages of sleep and you become less responsive to sensory stimuli so when you get down here into deep sleep you’re less responsive. So this is the one that you see slumped in the corner of the circle line then dribbling and all bets are off (audience laughter). And that’s deep sleep and that’s the stuff that makes you feel better—it is the deep sleep that makes you feel restored and vital and healthy.*

### A typology of medical narratives

Using an iterative, analytic induction approach the author developed a classification of the types of narratives that lecturers were using across the three lectures (see Table 1). The categories are based primarily on the content/subject matter of the narratives. Nine distinctive categories finally emerged which stood up to testing throughout the three lectures. This typology, while unlikely to be exhaustive, appears to have face validity from interviews with lecturers and students who seemed to understand and acknowledge the categories proposed. It attempts to capture the predominant categories of narrative [based on content] that lecturers told in their lectures about basic science. It also provides a framework within which to discuss the impact of narratives on the learning processes.

### Impact of narratives on learning

Key themes emerged from the data which echo the theoretical benefits of narrative learning—I will try to highlight these links throughout. Some themes apply to many or all narrative types; others are predominant features of one or two categories (see Table 1). I will illustrate findings with excerpts from interviews with lecturers and students.

### Meaning—making, [engagement, understanding, relevant context]

Both lecturers and students often talked about narratives and stories improving understanding and helping students to make sense of the material. Two particular themes emerged from the interviews: *engagement* with the material, and providing a *relevant context* for the audience.

This excerpt describes the students’ and lecturer’s perspective on a story about the history of Laudanum; they all highlight its power to engage and hold attention by providing a context for learning:

***Interviewer: And then the last one was about Paracelsus, the Swiss—German alchemist. Do you remember that story?***

*R5: Yeah, was that the tincture of opium and alcohol?*

*[Someone says: Laudanum].*

***Interviewer: So, that was a little story about Laudanum.***

*Student R4: … I really like the history of medicine as a personal interest, so bringing it into the lectures helps me to hold my attention, and also it gives you a greater scope of where it’s come from—how far back Opium goes. So, you can see its part in the big picture; I find it interesting but that’s just my opinion.*

*Student R5: I suppose they put it in there to engage you at the beginning, so you’re more likely to listen through the lecture.*

Lecturer 2 also highlighted the importance of relevant narratives to engage an audience, and to provide a “hook” using material that also interests him:

*L2: I mean I am a sort of frustrated historian I think so I will give them the historical context—it’s just interesting and I quite like it as a starting point for a lecture… lecturing is part engaging isn’t it? Once you have got your audience engaged then they are more likely to listen to you.*

The themes of engagement and providing a relevant context for learning also both emerged in discussions about the story in Lecture 1 about someone falling asleep on the tube (see earlier). Lecturer 1 talks about it as a “hook” to engage audiences, but also as a way to provide a context relevant to the students’ everyday lives:

*L1: Everybody loves the tube sleep.*

***Interviewer: So what, what do you think they would get from that? Why do you tell it?***

*L1: Because I inherently think it’s really interesting, because you sit in the tube, watching people. I do, anyway, when they go, you know, nod off. And I love it! It’s a kind of hook, isn’t it, so it establishes your credibility, and it allows them to ease into the lecture. You know, it kind of gets them going.*

*I mean, at a personal level I just think well you know, most of them are going to have travelled on the tube, and most of them will have experienced falling asleep. And most of the people have done that head bobbing thing.*

From observations of the lecture it appeared that students were engaged during the story; there was some laughter and chatter and heads were looking up at the lecturer. In subsequent interviews with the students, they all clearly remembered the story without prompting, but also agreed that they found it engaging and relevant and felt that because of those features it helped them to learn and understand:

***Interviewer: Anything else that you can remember from the lecture?***

*Student R5: Yeah, I can remember about the different levels of consciousness, as you fall asleep. So it’s like, the people who are just sort of asleep, they’ll lose muscle tone in their neck. But then she said, they’ll hear their station name, and then cause they’re only a little bit asleep it’ll wake them up. And the people who were so far gone, you could just watch them go to the end of the tube line. So I can remember stuff like that,*

***Why do you think she told that particular story?***

*Student R3 Erm, I think the whole tube story is relevant to all of us as students in London, it’s like, we’ll all remember it because firstly, like people laugh because you can apply that situation to, probably to ourselves and to friends and things, so you will remember it. And you can then put that story into your own context, if that makes sense?*

*Student R3: It’s more engaging. It was more applicable to us to use.*

The themes of engagement and providing relevant context for learning applied particularly to “story” narratives, driven by characters and struggles and goals (as illustrated by the two examples above); they were less often raised by students and lecturers in discussions of other narrative genres. These learning themes also seemed to appear more often in relation to narrative categories which provided particular relevance for the students—real world narratives that resonated with their existing experiences (eg the Tube Story) and narratives related to clinical cases, both real and hypothetical:

*Student; Yeah, I think in contrast to the narrative stories where I feel like I’m not relating to them and tend to tune out during those narratives, when it’s a clinically relevant story, like ‘this patient shows up in A&E and they have this’; I think I’m more likely to be paying attention to that, when it’s directly clinically relevant, because as soon as you brought that up I remembered almost verbatim what he said.*

### Professional identity development

Another prominent narrative theme which emerged from interviews was around professional identity. The sub-themes here were about professionalism (particularly professional attitudes), authenticity and trust, becoming an expert, and the fallability of science:

*L1: Well, I think that one of the things that is important, is that we actually don’t know where the site of respiratory control is in there. And I think it’s shocking, actually, that we don’t know that. …what you are getting over is, we don’t know it all.*

***I: Mm. And that’s an important message as part of your lecture?***

*L1: Well I think for me, that would be, um, exciting actually. Because it would mean that it’s not all in textbooks.*

Again, students got the message:

*R4: …she said something about it was very recent that we’d started to study breathing and things like that. Erm, partly due I think to technology and being unable to, but partly that it’s very hard to monitor, obviously, unless you’re asleep, it was something along those lines.*

All these themes have resonance with narrative learning theory in terms of using narratives to develop identity (in this case as a professional doctor), and in social terms (in terms of accepted professional behaviours, and the social enterprise of medical science). In Wenger and Lave’s theory of communities of practice , students in lectures could be seen as being “legitimate peripheral participants” of the professional medical community, engaging in joint activities and discussions, building relationships that help them learn from each other to develop a common professional identity [32]. From this research, it seems that narratives are being used in basic science lectures to fulfil this role in various ways, and lecturers and students acknowledge this.

### Memory

Memory was a strong theme which emerged from both lecturers and students. Students felt that narratives—again particularly the “story” genre—helped them remember information better. They often mentioned that stories helped them remember because they were engaging, but also that they were often specific examples relating to a particular person:

*R4: If you can remember a patient it’s always easiest, and you remember what the patient went through, and you can actually find that you remember a lot of the symptoms, the signs, the diagnosis, how the doctor diagnosed it, the treatment, and things like that. Just by relating it to this one, like person, and what happened to them. I remember all these little details that if you’re just remembering lists, actually it’s a lot harder to remember that.*

Students also saw narratives as memorable because they clearly outlined the relevance for students in their future work. A story about opiod overdose in A&E for example:

***I: Do you remember that?***

*[General agreement].*

*R3: It’s probably one of the clearest stories that I remember him saying, cause that’s useful, and that’s something we would need to remember in an emergency situation.*

The focus on memory appears to back up the theoretical links between the use of narrative and memory, and it is particularly interesting that it was the “story” genres of narrative, with their character driven plots, struggles and goals, that provoked the most discussion around memory. There were however several instances where students would volunteer what the author had classified as “stories”, unprompted, as the most memorable part of the lecture.

### Empathy/compassion

Several narratives—especially in the *real world experience* and *clinical case* categories—drew comments about empathy and compassion. Lecturer 1 particularly, told several narratives which seemed to be promoting the patient’s point of view, encouraging students to step inside a patient’s shoes. A story in Lecture 1 about a patient with locked-in syndrome triggered some strong reactions in some students:

*R1: I remember her saying this, and me thinking, oh my God that would be a horrible situation to be in.*

***I: You were thinking that?***

*R1: Yeah I remember her telling us about locked-in syndrome and me thinking that it would be such a horrible thing to experience.*

Another story, about sleep apnoea, encouraged students to imagine what it would be like to be woken every few minutes by their mobile phone going off…

*R4: Maybe just realising what it’s like for a patient in these conditions. It’s relating it to something you’ve probably never actually experienced it, but you could almost imagine more realistically what it’s like. It’s like with the mobile, it is annoying being once or twice, whereas if it’s every minute you can start to imagine going, hang on, whereas these poor people, nothing, it’s all about relating it to how you … it’s enabling you to empathise with the patient isn’t it.*

This is an example of a finding which was originally derived from observation and analysis of the lecture transcript, but which was later validated by asking the students and the lecturer herself if she agreed with my analysis. Before it was mentioned to her, the lecturer was unaware of the patient perspective nature of the narratives she was using:

*L1: And what you’re demonstrating to me, which I couldn’t have told you if you hadn’t read them all out, is that these narratives frequently come from a patient perspective.*

The promotion of empathy and compassion is a strong theoretical benefit of narrative pedagogy—these findings suggest that even in basic science lectures, this aspect of narrative learning is indeed at work. Students and lecturers both acknowledged that the value in many of the narratives used was in promoting a patient perspective.

### Reflection on practice

Although not a formal element of the lectures, reflection did come up in discussions. For example, lecturers talked about using narratives as punctuation marks in lectures, to give the students a rest, and time to reflect on what’s gone on before.

*L2: When you are telling a story, they are not going to take notes of a story—it’s obvious it’s a story and therefore they have to stop, and as soon as they stop writing then they are thinking. Then maybe you’re hoping you’re engaging a different pathway so that maybe now they think okay now I understand better because I have actually stopped to listen.*

Students seemed to agree that this was another positive feature of narratives in lectures:

*R6: Just putting a break to the number of lists and facts.*

***I: So, almost like punctuation, and a breathing gap.***

*R6: Yeah, exactly.*

*R1: It’s a really good moment for consolidation, in that sense, so, we’ve had the dry list at the start, and then if you get an anecdotal story or whatever afterwards it’s not only a breathing space but you can sit back, you’re not taking notes for a moment, but it all puts into context what you’ve just written down.*

### Lecturer and student attitudes to the use of narratives and stories in lectures

Students, on the whole, were very receptive to the idea of lecturers using narratives or stories in their science lectures, particularly given their view of medicine as a distinctly human endeavour. Students also seemed to distinguish between the (legitimate) use of narratives to aid student learning for a clinical context, and the (discouraged) use of stories in formal scientific discourse:

*R3: Stories are good from our perspective, the ones that relate to general day to day life on the ward, cause they teach you what to look out for and put in context what you need to know. But you’re aware that if you were gonna publish a study or something you’d leave out the anecdotal evidence because it’s not viewed as scientific. So, I think those kinds of stories are good in the right context, so to speak.*

But a minority of students had negative comments to make about the use of narratives in lectures. The three main themes here were that narratives/anecdotes may be more to do with entertainment than learning, that they are too often irrelevant asides (and particularly not focussed on passing exams), and that too many of them can become boring:

*R2 I find the anecdotes interesting, but I don’t think they necessarily help me to learn the material any more. They’re interesting, but I think they’re an aside. So, personally, I don’t think having them there improves my understanding.*

*R3 …sometimes there can literally be just too many referring to, not the actual lecture itself but all the kind of anecdote stories and things, and then you're not going to remember them, and you just lose interest as well.*

The lecturers were also open to using narratives in their lectures, and felt quite passionately that their “non-scientific” nature did not detract from their value. One didn’t accept my suggestion that narratives might be perceived as rather “fluffy” in a scientific institution:

*L2: No, I like fluffy so I don’t have a problem with the slightly less scientific. I don’t even mind telling a story that can be scientifically irrelevant or even inaccurate sometimes if it’s making a point.*

But lecturers were aware that not all students were well-disposed towards the use of narratives in lectures. Lecturer 2 wondered whether this was because some students simply don’t learn that way:

*L2: There are the students who actively rebel against it and get a bit annoyed and think it’s a waste of time. Whether there are particular types of learners who need that straight narrative to maintain concentration, and then by telling a story you are actually distracting them and therefore they disengage with the lecture; I guess that’s the only downside*.

I was interested too in whether the lecturers used narratives deliberately, or were even aware of the fact that they were using narratives and stories at all. Lecturer 2 was aware that he was using certain types of narratives, and did use them deliberately:

*L2: Yes, definitely; so historical stuff I guess just for consistency. Personal stuff and historical stuff you can apply in a lot of instances and then outside of that you are trying to find anything real world that you can apply to your teaching just to try and give it more interest and get them to think about it.*

But he was less aware that he was telling some other categories of narrative:

*L2: …it’s even the things like the opioid overdose without necessarily thinking of it as a story so that’s interesting. I guess that kind of thing it might make you more inclined to try and make your stories more clinically relevant more often…*

Finally, Lecturer 1 felt that while she had no problem with the concept of using narratives in teaching and learning, she did perceive a problem with the actual words “story” and “narrative”:

*L1: I think it’s the word story that’s quite tricky, because it’s kids and it’s bedtime reading. And so it doesn’t have the same level of impact than if you can call it something else. We need to find a more sophisticated name for it.*

## Discussion

From the findings presented, it appears that lecturers are using many narratives in their basic science lectures – even when they don’t think they are. Some of them are using narratives purposefully, but usually only on certain topics such as the history of medicine. The typology describes a variety of narrative themes, from clinical cases (both real and hypothetical) to narratives about patient experience or their own professional careers. Certain categories of narrative seem to carry particular shared meanings between students and lecturers, and these echo the theoretical benefits of narratives described in the literature [2, 5, 6]—for example, clinical case narratives and real world experience narratives often conveyed messages about professional identity—narrative theory holds that we create our identities—including professional identities—by storying them [2]. Patient narratives were often interpreted as promoting empathy or awareness of others—another theoretical benefit of narratives in learning, encouraging the listener to put themselves in another person’s shoes and to enter worlds which are otherwise closed to them [5]. In addition certain categories were associated with particular aspects of narrative learning—character-driven stories in particular seemed to be better remembered by students, especially narratives with relevance to their own professional context (for example, clinical cases).

Whilst students felt that narratives or stories would not be appropriate to use in a formal scientific research paper, they were mostly receptive to the idea of using narratives for learning in lectures. A minority of students though, felt that narratives could be an irrelevant distraction, particularly if not related to learning objectives to be tested in exams. This may reflect different learning style preferences in different students. It is interesting that lecturers often underestimated the number and range of narratives or stories they were telling; this in itself might reflect a reluctance to consider them as useful learning tools. However on direct questioning, lecturers were even more positive than students in their attitudes to using narratives and stories, although there was some feeling that the word “story” was problematic as it had connotations of childishness. There was little sense, as some have suggested, that the use of stories and narrative in medical education is undervalued, even maligned [33].

Most students (but not all) felt that narratives can be a valuable learning tool. Several key parts of the learning process came to light in this study which resonate with the existing literature. Both students and lecturers highlighted narrative’s value in providing a relevant context for them—by drawing them in to the story and engaging them, stories seemed to help them understand the material better. A key step for any learner—including medical learners—is to create meaning from what they experience; to make it make sense. Meaning-making is very closely linked to comprehension or understanding—a learner must comprehend before he or she can create meaning. There have been many studies of patient’s illness narratives which add empirical weight to the theoretical centrality of narrative in meaning-making. Hanninen, for example, found that patients with addictions were unable to promote self-recovery until they were able to construct a successful narrative of their addiction [1]. But another important pedagogical spin-off of meaning-making is engagement. Narratives and story structure may help to *engage* learners by offering a connection with existing knowledge and experience, and by making the unfamiliar seem familiar. For example Holt and others [34, 35] have shown that story structure can help learners call up existing banks of knowledge, and can make new information seem relevant. In particular, students found stories about clinical situations they are likely to encounter in their careers engaging and relevant. This supports the concept of narratives being a key element of the experiential learning cycle—and as MacNaughton has suggested, an effective way of vicariously acquiring information about clinical cases [8].

Students and lecturers focused a great deal on the value of narratives in promoting memory, something which is likely to help in the development of useful illness scripts to be retrieved later on in their careers. Clinical case narratives seemed the most relevant category of narrative in this regard. Hunter, from her observational studies in educational settings within hospitals, has postulated that narratives may be a useful way for medical students to retain information [2]. Fernald’s study of eighty undergraduates suggested that they found stories more likely to be remembered than formal book or lecture descriptions [36]. However this line of argument doesn’t address the possibility that different learners may have different preferences in learning styles—while some may favour stories, others may prefer to learn through non-narrative approaches such as lists or diagrams [37]. There is, though, increasing evidence from the field of applied cognitive psychology that narrative skills are central to long term retention and retrieval of personal memories. Studies have shown for example that children’s ability to construct coherent narratives is linked to their ability to provide more information about events [38]. Narrative learning theory highlights both memory and engagement as potential strengths of narrative structures. Classical stories particularly are memorable because they involve the listener in the actions and intentions of the characters. They demand active meaning-making; we have to fill in any gaps and create as well as discover meanings within stories [11]. Stories engage learners by involving them and stimulating an empathic response – the vivid details of characters and their motivations draw us into a story and promote perspective taking [11]. In this study, lecturers highlighted more the value of narratives in promoting empathy, and offering insights into the professional world of medical science, although students too could see this when it was suggested to them.

The literature suggests that narratives are central in professional identity development, passing down cultural values and norms of professional behaviour through generations of doctors [39]:

*In medicine, parables often start with “I had this great case” or “When I was an intern.” What ensues is a story about a fascinating medical case with a moral about what it means to be a doctor*.

Based on her scholarly studies of narratives in medicine, Hunter identifies an important role of narrative in aiding socialisation and professionalization of medical learners [2]. Narrative learning theory holds identity development and social aspects close to its core, so in theoretical terms at least, it is easy to see how narratives could act as a vehicle via which issues of professional identity and culture might be shared. This study adds weight to these ideas—lecturers often choosing to tell stories about professional culture, careers, and the fallability of science.

Finally, of all the narratives identified, it was the ones categorised as “stories” with a character-driven plot, and some emotional struggle towards achieving a goal, which provoked the most discussion and positive remarks about nearly all the aspects of learning I have highlighted. This resonates with Haven, Bruner, Ricoeur and others [40–41], that there may be something particularly powerful about character-driven “stories” as a genre of narrative as a learning tool.

### Limitations

There are several limitations to this project. For practical reasons it was only possible to observe three lectures (and therefore three interviews and focus groups), which raises the possibility of various biases; this may not have captured the full range of narrative categories that a larger sample of lectures would have demonstrated, and the sampling of lecturers may well have favoured those who use narratives more often than average. Equally, by disclosing the focus on narratives in the invitation to students, there may be a selection bias within the focus groups. However, neither of these issues would necessarily bias findings with regard to *how* stories are used in learning, or the themes they cover.

There may also have been an important observation bias, where participants behave differently purely as a result of being observed. As a result of being transparent about the narrative nature of the study it is possible that lecturers may have included more narratives than they normally would. However, they were asked directly if they had included more narratives than usual; all denied this was the case, and the narratives were ones they used every year in the same lecture. In addition, this would not affect the analysis of meaning or elements of learning process – only the number and types of narratives used.

Students may also have been reluctant to criticise their lecturers. Students may have felt under pressure from the author as a senior member of Faculty to be enthusiastic about narratives (although the author had not taught any of them and made clear that honesty was encouraged). However, it was reassuring that several students felt quite happy to make critical observations about both lecturers’ styles and their use of narratives or stories. It is also possible that in choosing audio over video, the study may have missed some important opportunities to observe key audience reactions or contextual features of narrative learning, though field notes included visual observations.

## Conclusions

The findings support existing literature in this area which suggests that narratives may be a useful tool for learning in medicine. This study suggests that narratives tap into several key learning processes including providing a relevant context for understanding, engaging learners, and promoting memory. For medical students in basic science lectures, narratives or stories may be particularly relevant in promoting the more humanistic aspects of medicine, including professional identity, and empathy. More research is needed to provide evidence of the impact of narratives or stories on learning outcomes for students. However, for lecturers or faculty interested in using narratives—especially stories—as a teaching tool, this study may offer greater awareness of their potential and some guidance in how to use them in a more focused way.

## Glossary

### Narrative

My working definition of narrative, derived from a wide literature, including the fields of narratology, literary criticism and linguistics is:

*A sequence of events connected together in a way that gives them meaning.*

### Story

Within this over-arching definition of narrative sit many different genres of narrative ([31], P 231). “Story” is a particular type of narrative; all stories are narratives, but not all narratives are effective stories. Stories have recognisable elements such as a protagonist, conflict, climax and resolution. This is Scholes’ widely-used interpretation of story:

…*a telling or recounting of a string of events with at least three basic elements: 1) a situation involving some predicament, conflict or struggle, 2) an animate protagonist who engages with this situation for a purpose, 3) a plot during which the predicament is somehow resolved*

([42], p. 59)

For simplicity I adopted Haven's definition for this study: "A detailed, character-based narration of a character“s struggles to overcome obstacles and reach an important goal" [43].
